# Editorial: The Association Between Greenness, Health, and Well-Being in Urban Environments

**DOI:** 10.3389/fpubh.2021.732876

**Published:** 2021-08-23

**Authors:** Zhonghua Gou, Ye Liu, Yi Lu, Wenjie Wu

**Affiliations:** ^1^School of Urban Design, Wuhan University, Wuhan, China; ^2^School of Geography and Planning, Sun Yat-sen University, Guangzhou, China; ^3^Department of Architecture and Civil Engineering, City University of Hong Kong, Hong Kong, China; ^4^College of Economics, Jinan University, Guangzhou, China

**Keywords:** greenness, health and well-being, urban environments, green space, urbanization

The collected articles address a wide range of critical issues, such as accessibility, environmental satisfaction, air pollution, social-cultural values, and ecosystems, which link urban greenness and urban residents' health and well-being in multifaceted perspectives.

Several articles in this special issue focus on how older people's mental health and well-being are affected by green space. Zhou et al. contributed an important study to this special topic by clarifying the link between green space and older people's mental health and well-being. They used multi-scale data including both remote sensing and questionnaire surveys. Their study argued that there is a critical element linking green space and mental health, that is social interaction. Therefore, planning and designing green space for social interaction should be included in the study of green space and health. This special topic also includes a study looking at the older people from diverse cultural backgrounds. Gao et al. investigated older Chinese immigrants in Australia and found out their green space preferences. The physical activity and good accessibility to green space have been valued by Chinese immigrants, which has important implications for designing inclusive public space. Zhai et al. contributed another empirical study about the link between older people's walk behaviors and park features. Their study identified a series of park characteristics that can be used to create walkable parks and outdoor spaces. Shi expanded the enquiry of green space beyond the psychological well-being; she argued that green space also means environmental comfort, hygiene and security to the senior citizens in Hong Kong. Based on her studies in public housing in Hong Kong, she identified some critical design elements to enhance older people's perception of security, hygiene, and environmental comfort in public green space.

Promoting equitable access to green spaces is crucial to the construction of sustainable and equitable cities. He et al. examined the extent to which different types of green spaces (urban parks and other urban vegetation) were provided to the total population (referring to as horizontal equity) and different social groups (referring to as vertical equity) in an equitable manner in Wuhan, China. Their findings suggested that the inequity of public parks was more intense than mixed and woody vegetation, and that urban green space supply was highly skewed toward the inner and outer areas. Cheng et al. took a dynamic community deprivation consideration and looked at the accessibility issue from the perspective of transport modes and traffic networks. Their results highlighted the socioeconomic factors underlying the disparity of accessing to the urban green space and called for political attention to the inequality of green space accessibility.

This special topic also collected articles focusing on the physical health benefits of greenness, such as reducing risks of air pollution, skin disease and obesity. Using air quality monitoring data from 285 cities in China, Wang et al. explored the effect of green coverage (GC) on PM2.5 concentration during peak hours. They found a considerable heterogeneity across cities regarding the marginal effect of GC on the mitigation of PM2.5 concentration. They also indicated that government expenditure on urban maintenance could mitigate air pollution through economic development. For this reason, policymakers from different regions were advised to use different combinations of policies to mitigate air pollution. Acne vulgaris is a commonly seen skin disease that has a considerable impact on the quality of life. Yet the complex association between environmental factors and the occurrence of acne is unknown. Yang et al. provided a comprehensive review of literature on the impacts of individual and contextual factors on the occurrence of acne. They further indicated the possible pathways linking built environment to the pathogenesis of acne and called for more empirical studies to test their suggested pathways. Xiao et al. used a deep convolutional neural network architecture to extract eye-level information from Street View images to capture the urban vertical greenness level and identified the view-based green index that might have an protective effect on body weight.

The perception and satisfaction of green space has been investigated for different groups of users. He et al. explored cyclists' perception and experience of using greenway in Wuhan. The research addressed the cyclists' landscape imagery and their visual perception elements that should be well-understood in designing greenways. They found four features that contribute to a positive cycling experience: continuous cycling paths, high security awareness, open landscapes, and rich human activities. Chen et al. took a disparity perspective to study the perceptions of urban green space between residents and tourists nearby East Lake in Wuhan and identified the underlying difference of perceptions between the two groups. Qiao et al. used an individual survey from Shenzhen metropolitan areas to document the mediating role of satisfaction on the relationship between green space and urban residents' mental health. Mao et al. defined cultural ecosystem services in a broad sense that include green space to enhance mental health, social belonging, group identity, and social integration. They surveyed 40 communities and their residents in Zhengzhou, China to demonstrate the compensation effect of green space in response to the lack of nearby parks. They also addressed the important role of natural vegetation in urban residential communities' cultural ecosystem services.

This special topic has attracted contributions on a broad spectrum of dimensions of the green space availability issue. The topic has inspired a dialogue between different disciplines that is crucial to understand the complex relationship between availability of green spaces and public health. The sample size is becoming larger and more extensive, to capture the complex urban environments and populations. The link between greenness and health and well-being remains inconclusive, awaiting new methodologies, techniques and measurements. Another limitation is that all contributions are from studies conducted in China except one, in Australia on Chinese immigrants. An obvious question is whether findings from such studies could be applied to other settings such as Europe or the U.S. It might not be necessarily the case. However, the studies covered in this special topic show promising evidence to support that urban greenness promotes the health and well-being of various population subgroups in various contexts.

Last but not least, the editorial team would like to thank every author and reviewer for their contribution to this special topic.

## Author Contributions

All authors listed have made a substantial, direct and intellectual contribution to the work, and approved it for publication.

## Conflict of Interest

The authors declare that the research was conducted in the absence of any commercial or financial relationships that could be construed as a potential conflict of interest.

## Publisher's Note

All claims expressed in this article are solely those of the authors and do not necessarily represent those of their affiliated organizations, or those of the publisher, the editors and the reviewers. Any product that may be evaluated in this article, or claim that may be made by its manufacturer, is not guaranteed or endorsed by the publisher.

